# Scaphoid Plate Osteosynthesis in Complex Fractures and Wrist Trauma: A Case Series

**DOI:** 10.7759/cureus.45067

**Published:** 2023-09-11

**Authors:** Chai Jiun Liau, Siew Khei Liew, Syahril Rizal Arsad, Rashdeen Fazwi Muhammad Nawawi, Jeremy Prakash Silvanathan

**Affiliations:** 1 Hand & Microsurgery Unit, Orthopedics Department, Hospital Selayang, Selangor, MYS; 2 Hand & Microsurgery Unit, Orthopedics Department, Hospital Kuala Lumpur, Kuala Lumpur, MYS; 3 Hand & Microsurgery Unit, Orthopedics Department, University Putra Malaysia, Selangor, MYS

**Keywords:** nonunion, wrist trauma, complex, scaphoid plate, comminuted, scaphoid fracture

## Abstract

Introduction

Treatment of scaphoid fracture is challenging due to its unique blood supply and geometry. Traditionally, a headless compression screw is the standard treatment for unstable scaphoid fracture. Some fractures are complex, for example, comminution with bone loss. A scaphoid plate is an option in these difficult fractures providing adequate rotational stability.

Aim

To share our experience in using scaphoid plates in complex wrist trauma and comminuted fractures.

Method and material

Complex wrist trauma involving scaphoid fractures that were comminuted and multi-fragmentary fractures treated with plate osteosynthesis were retrospectively reviewed between July 2019 and September 2021. Patient demographic data, preoperative radiographs, CT scans, pain, wrist range of motion, and fracture union rate to union were reviewed. Quick Disabilities of the Arm, Shoulder, and Hand (QuickDASH®) score was recorded at 1-year postoperative follow-up.

Results

Nine patients associated with complex wrist trauma were included in this case series. The mean follow-up was 2.5 years (13-30 months). The union rate was 100%. The mean arc of motion was 105° (95-110°). QuickDASH® score was 19.96 at 1-year postoperative follow-up. Four patients had good outcomes, four satisfactory, and one poor outcome. One hardware complication was observed which was the impingement of the plate proximally over the articular surface of the distal radius.

Conclusion

A scaphoid plate is a reliable option for treating complex and difficult fractures. It provides adequate stability, especially in comminution, bone loss, or multi-fragmentary fractures which are not amendable using other fixation methods. We recommend the expansion of plate osteosynthesis beyond scaphoid nonunion into complex wrist trauma.

## Introduction

Scaphoid fractures are the most common carpal bone fractures. Due to its unique blood supply and geometry, treatment is quite challenging. Treatment of the simple unstable scaphoid fracture using a headless compression screw is considered a standard acceptable treatment after Herbert and Fisher introduced the Herbert screw in 1984 [1,2]. Despite the treatment, some fractures go into nonunion, and subsequently collapse, causing dorsal intercalated segmental instability (DISI) deformity and, lastly resulting in degenerative arthritis. Salvage procedures such as proximal row carpectomy and four corner fusions are inevitable if arthritis has developed.

Some of the scaphoid fractures are complex with bone loss, significant comminution and some of them are recalcitrant nonunions that were previously treated with screws. These complex scaphoid fractures are difficult to treat with screws. The scaphoid plate comes in as an option to fix these difficult fractures on the principle of providing buttress support and rigidity and also giving good rotational control of the proximal and distal poles [3].

The idea of scaphoid plating is not new. It was first described by Ender in 1977 to treat scaphoid nonunion with a hook plate where one screw in the distal pole and a staved hook in the proximal pole [4]. In recent years, a newly developed volar locking plate has been designed to treat recalcitrant scaphoid nonunion and difficult fractures [3,5]. However, there are very limited reports in the literature on complex wrist trauma and complex scaphoid fractures that are treated with a scaphoid plate.

The aim of this study is to share our experience in using the scaphoid plate in complex wrist trauma cases with comminuted and fragmentary scaphoid fractures.

## Materials and methods

We retrospectively reviewed scaphoid plate osteosynthesis in complex wrist trauma performed in our centers between July 2019 and September 2021. 

A total of nine patients were included in this case series. There were six trans-scaphoid perilunate fracture dislocations with a combination of other fractures such as fracture hamate, triquetrum, pisiform, distal end radius, ulnar styloid, and scapholunate ligament injury. Two were comminuted scaphoid fractures and one was an open Galeazzi fracture with carpal bone fractures. All cases were fixed with a scaphoid titanium variable angle locking plate of size 1.5 mm (Medartis AG, Austrasse, Basel, Switzerland). 

Preoperative radiographs and CT scans were reviewed. Clinical assessment during follow-up was assessed for pain score and wrist range of motion. The arc of movements of flexion and extension were extracted at the last follow-up. Radiology assessment for fracture union using radiographs was done every follow-up visit. Fracture union was defined as no fracture line on 3 cortices radiologically. Radiograph PA, lateral, semi-pronated, semi-supinated, and ulnar deviated radiographs were done. Follow-up duration was minimal at a mean of 13 months postoperative (13-39 months). Quick Disabilities of the Arm, Shoulder, and Hand (QuickDASH®) score (0 = no disability; 100 = total disability) was scored at the end of the 1-year follow-up to evaluate the wrist function. 

All surgeries were performed by two senior consultants in the hand and microsurgery unit and the orthopedic department. All patients were put under general anesthesia in a supine position with their hands on the arm table. An arm tourniquet was applied. The volar approach between the radial artery and flexor carpi radialis (FCR) was used to expose the scaphoid [6]. An incision was started about 4 cm proximal to the wrist crease and curved to the distal scaphoid tubercle distal to the wrist crease. The superficial fascia was opened and then the flexor carpi radialis sheath was opened and retracted ulnarly. The superficial palmar branch of the radial artery was identified and ligated. The palmar wrist capsule and radioscaphocapitate ligament were partially incised for exposure of the entire scaphoid. The incision was extended proximally for distal end radius fixation if there was a combined procedure or if the bone graft was needed. The fracture was reduced and held with K-wires. Humpback or rotation was corrected. The distal radius bone graft was harvested if needed to reconstruct the waist length in those with comminuted fractures. Precontoured scaphoid plate 1.5 mm (Medartis) was placed volarly at the center of the scaphoid. Three screws were inserted each proximal and distal fragment. Restoration of length and shape was checked intraoperatively and fluoroscopically. 

Postoperative, the wrist was immobilized with thumb spica slab with free metacarpophalangeal joint of the fingers for 4-6 weeks due to the complexity of fractures and combined injuries. A removable splint was applied after 6 weeks and active range of motion exercise was commenced after 6 weeks. Follow-up interval was at 2 weeks, then monthly, till bony unions were observed on radiographs and at 6 and 12 months. Radiographs were taken in every follow-up.

## Results

A total of nine patients who underwent volar plating of scaphoid between July 2019 and September 2021 were included (Table 1).

**Table 1:** Patient demographic data PLFD = periluante fracture dislocation; SL = scapholunate; MVA = motor vehicle accident

Patient	Age	Sex	Side	Diagnosis	Mechanism of injury	Scaphoid location
1	27	M	Left	Transcaphoid transcapitate PLFD, fractures hook hamate, triquetrum, ulnar styloid	MVA	Waist
2	22	M	Left	Transradial transcaphoid PLFD	Sports injury football	Waist
3	47	M	Left	Open Galeazzi fractures, scaphoid and triquetrum fractures	MVA	Waist
4	27	M	Right	Transcaphoid PLFD	MVA	Waist
5	34	M	Left	Scaphoid comminuted fracture	MVA	Waist
6	25	M	Right	Transradial, transcaphoid PLFD, fracture ulnar styloid	MVA	Waist
7	32	M	Right	Transcaphoid PLFD, fracture distal end radius	MVA	Waist
8	38	M	Right	Transradial, transcaphoid PLFD, fractures capitate, hamate, triquetrum, pisiform, SL ligament injury	MVA	Waist
9	31	M	Right	Scaphoid comminuted fracture	MVA	Waist

All the cases were secondary referral cases from other hospitals. All the fractures were acute trauma. The time of injury to fracture fixation was 2-6 weeks. All patients were males with a mean age of 31.4 (22-47 years). Five on the right hand and four on the left hand. 

Eight patients were involved in motor vehicle accidents and one was involved in a sports injury. One patient had previous surgery done in another hospital which was external fixation and K-wires, plating radius, and triangular fibrocartilage complex (TFCC) repair. 

All the scaphoids were fractured at the waist and the fractures were fragmentary. Eight were closed fractures and one was an open fracture. Two patients had bone grafts harvested from the distal radius. Seven patients had combined procedures of fixation for other fractures while two patients had only scaphoid plating.

**Figure 1:**
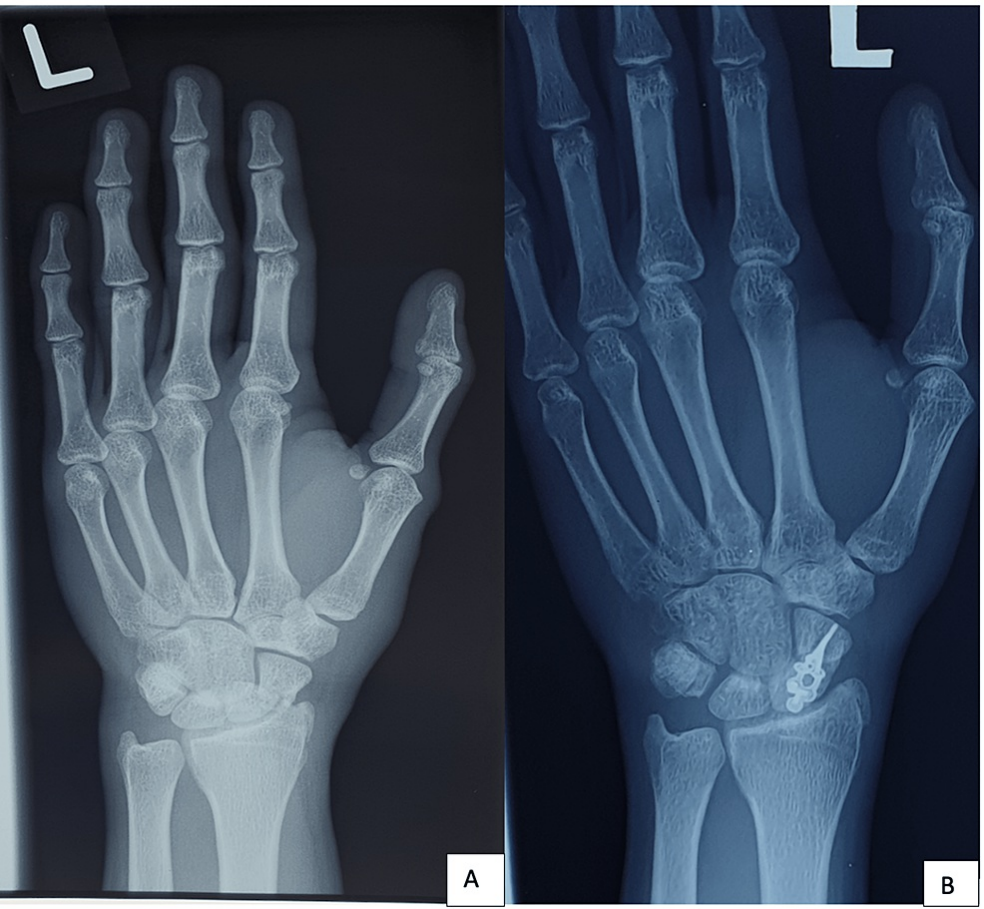
A. Preoperative radiograph: transradial transscaphoid perilunate dislocation; B. 1-year postoperative radiograph

The mean follow-up was 2.5 years (13-39 months). The mean time to fracture union was 4.8 months (4-6 months). All fractures (100%) were healed (Figure 1). 

The mean arc of motion was 105° (95-110°). QuickDASH® score was at the mean of 19.96 at 1 year post-surgery. Four patients had good outcomes, four were satisfactory, and one had a poor outcome (Table 2).

**Table 2:** Functional outcome M = month; ROM = range of movement; QuickDASH = Quick Disabilities of the Arm, Shoulder, and Hand

Patient	Union time (M)	ROM arc (degree)	QuickDASH	Complication	Outcome
1	6	100	13.6		Good
2	4	110	15.9		Satisfactory
3	6	105	11.4	Impingement proximal plate	Good
4	4	100	27.3		Satisfactory
5	4	110	11.4		Good
6	5	110	25		Satisfactory
7	5	110	22.7		Satisfactory
8	6	95	40.9		Poor
9	4	110	11.4		Good
	Mean: 4.8	Mean: 105	Mean: 19.96		

**Figure 2:**
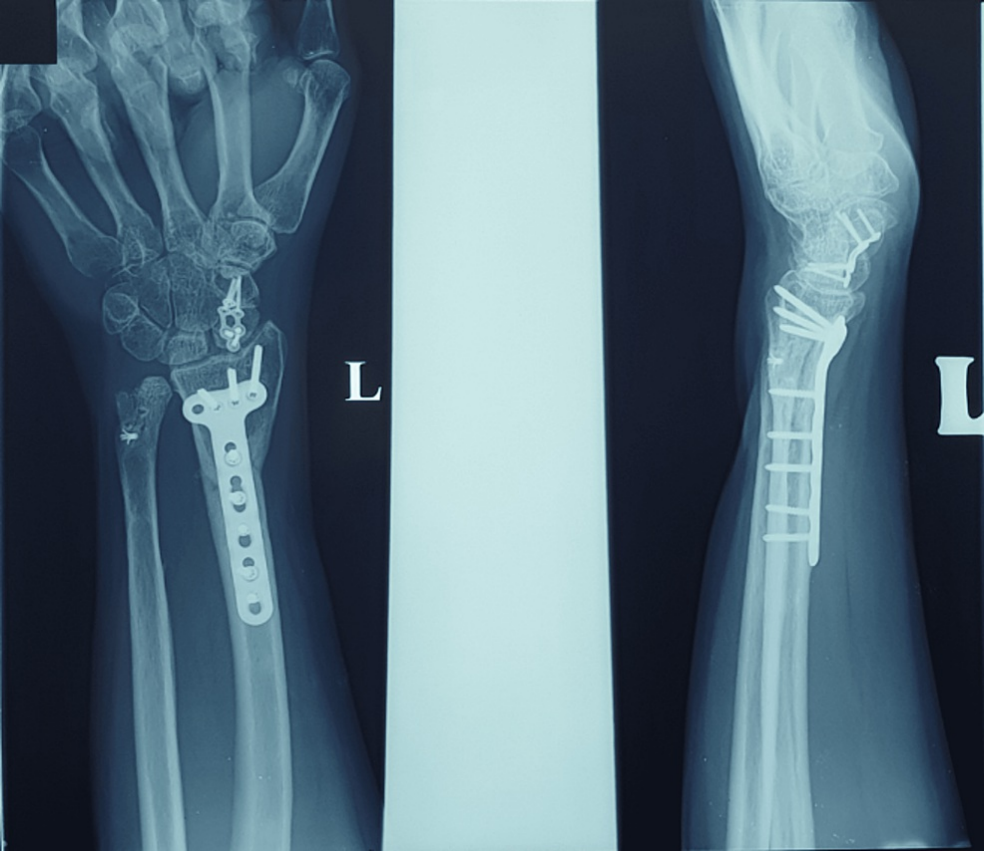
Radiographs showed hardware prominent and impingement of the proximal plate on the volar lip of the radius

We noted one complication from this case series. One patient had a hardware issue which was impingement of the plate proximally over the distal articular radius (Figure 2). 

The patient was asymptomatic and had a good range of motion and he refused to remove the plate. No hardware removal was done up to date. 

## Discussion

Treatment of acute scaphoid fracture traditionally with a single headless compression screw is widely an accepted technique since the introduction of the Herbert screw in 1984 [2]. It has been used for acute fracture and delayed union and nonunion cases. The success rate of the union with screw was up to 95% [7]. However, some fractures still went into nonunion despite screw fixation. The possible risk factors are loss of rigid fixation, malposition of the screw, inadequate reduction, or avascular necrosis of the proximal pole [7]. Fractures with bone loss and comminution are difficult to treat with a single compression screw to provide a rigid fixation and rotational stability. These types of fractures are difficult to achieve union and subsequent treatment will be demanding. 

The concept of scaphoid plating was first introduced by Ender in 1977 for the treatment of scaphoid pseudoarthrosis [4]. It was a beaked plate where the distal fragment and iliac bone graft were fixed with a screw and the proximal fragment with a staved hook. Removal of the implant is necessary because this implant was partly intraarticular. In recent years, a modern implant volar extraarticular scaphoid plate has been introduced (Aptus® Trilock 1.5, Medartis AG, Austrasse, Basel, Switzerland). This plate is pre-contoured, low profile of 0.8 mm, and consists of locking and nonlocking screw options. It also provides more buttress support and rigidity, especially in the presence of bone loss. The screws are variable trajectories and are theoretically able to control the rotation and angle of each fragment [8]. It is very useful in the reduction of humpback and flexion deformities. 

Biomechanically, locked plates convert shear stress to compressive stress at the screw bone interface. An angular plate system provides more rotational stability compared to a single compression screw fixation [9]. However, there was no difference between the two compression screws and the angular plate [9,10]. Quadbauer et al. showed that in scaphoid waist nonunion, the fracture healing was better with two compression screws than with a single compression screw and the plate had the highest union rate [11]. Locking plate was also superior for complex scaphoid fracture, especially in osteopenia bone, and had a greater load to failure compared with screw fixation [12]. It is believed that a higher union rate is achieved with a more stable fixation system. The scaphoid plate is preferably used in the treatment of dislocation or comminuted fracture and nonunion [9]. A biomechanical study of plate versus screw in segmental scaphoid fracture showed that plate and screw fixation demonstrate similar loads to failure, but plate fixation was superior for gap recovery after an applied load to failure [13]. Another benefit of a plate is that it can prevent the extrusion of cortico-cancellous bone grafts in cases of nonunion. It is difficult to hold the bone graft with a screw together with fracture fragments. 

In our case series, most of the cases were complex trauma with perilunate fracture dislocation, and the fractures were severely displaced or comminuted. Plating is biomechanically better than a screw in fixing these types of fractures. Our result showed that a 100% union rate was achieved with scaphoid plating. The result of the union was consistent with the literature, more than 90% [5,14-16]. A systemic review by Morgan et al. showed that scaphoid plating union rate 72-100% which included nonunion and trauma groups [17].

General indications of scaphoid plating are nonunion with bone loss and humpback deformity, revision surgery with failed previous surgery and central cavitation, acute waist fracture with significant comminution, bone loss, and acute unstable oblique waist fracture [3]. In our case series, we expanded its usage into acute complex wrist trauma.

The mean motion arc in our study was 105° whereas in other studies was 94°(Feliu et al.), 104° (Mehling et al.), 120° (Dodds et al.), 115° (Eng et al.), and 177° (Leixnering et al.) [5,8,16,18,19]. In a systematic review of a few articles, the mean was 119° [19]. The motion arc varies between studies. The possible reason is the variability of different clinical cases ranging from trauma to nonunion in the studies. In our case series, the range of motion was slightly reduced compared to the literature because cases were more complex with multiple fractures or wrist pathology that contributed to wrist stiffness. 

QuickDASH® was good in four patients who scored less than 15. Some are them were complex injuries with perilunate fracture dislocation. One patient had a poor outcome with the QuickDASH® score of 41%. This patient had the most complex wrist injuries with most carpal bone fractured and perilunate dislocation associated with scapholunate ligament injury. There was no hardware impingement in this patient. Interestingly, some perilunate fracture dislocations had a good outcome even though one of the cases had hardware impingement of the proximal plate. 

Most of the complications reported from scaphoid plating in the literature were impingement of the proximal plate on the volar lip of the radius, plate, or screw breakage. In one case series, authors reported high complications (10/15) including plated breakage, screw back out, and impingement [19]. Their result of scaphoid plating was not satisfactory. Whereas a systemic review of eight studies reported 21% of plate removal in 151 plated scaphoid nonunion [17]. Reasons for removal were impingement on the radial styloid, plate breakage and screw back out, and clicking or pain on maximal flexion [8,17-20]. Mehling et al. reported 66.7% removal plate because of impaction plate and protrusion screws [18]. 

Some studies recommended the removal of the implant at four months due to hardware complications [15]. Dodds et al. suggested placing the plate more distally to prevent the impingement [8]. Putnam et al. mentioned plate should not be placed beyond the concave-shaped area of the proximal pole, the "watershed line" similar to the distal end radius concept [21]. For proximal pole nonunion they cut off the most proximal hole with two screws inserted proximally. Eng et al. recommended checking the impingement intraoperatively and placing the plate more distal if there is impingement [16]. For more proximal pole fractures, they do not recommend plate fixation instead of a headless compression screw. 

We recommend a scaphoid plate in waist fracture but not in very proximal pole fracture. In a proximal pole fracture, proximal screws might not have good bone purchase which will affect the stability of the plate construct. The limitation of single plate size design is a challenge in smaller bone size or wrist in some Asian patients; a scaphoid plate might not be suitable as it might overhang proximally lead to impingement, and cutting off one of the screw holes might be needed. Surgeons should be cautious of these technical issues and anticipate hardware removal should any complications arise. However, the scaphoid plate provides excellent stability, particularly rotational stability, and as a bridging device in fixing more comminuted and complex scaphoid fractures. The result observed in this series albeit the complexity of wrist trauma was satisfactory in terms of union rate and range of motion. 

The limitation of this study includes a retrospective small sample size due to limited indication for scaphoid plating. Most of the scaphoid fractures can be treated with a headless compression screw. Secondly, most cases were complex trauma and involved other fractures that needed to be fixed at the same time or at different times. It makes the study less standardized and not comparable in terms of the outcome. It is difficult to assess the preoperative function because of the complexity of the fractures and immobilization. Lastly, we could not obtain a postoperative CT scan to accurately assess the position of the plate and screws due to limitation of resources. Assessment of union and consolidation would be more accurate and standardized with a CT scan. 

## Conclusions

A scaphoid locking plate is a reliable option for treating complex and difficult scaphoid fractures. It provides adequate stability, especially in comminution, bone loss, or multi-fragmentary fractures which are not amendable using other fixation methods. It is technically more demanding to perform scaphoid plating in complex fractures than a compression screw. Hence, surgeons should be aware of the possibility of hardware issues and the need for the removal of the plate should any complications arise. It is an excellent treatment option when screw fixation is not possible or failure of previous screw fixation. We recommend the expansion of plate osteosynthesis beyond scaphoid nonunion into complex wrist trauma.

## References

[1] Herbert TJ, Fisher WE (1984). Management of the fractured scaphoid using a new bone screw. J Bone Joint Surg Br.

[2] Herbert TJ, Fisher WE, Leicester AW (1992). The Herbert bone screw: a ten year perspective. J Hand Surg Br Aug.

[3] Wu F, Ng CY, Hayton M (2019). The authors’ technique for volar plating of scaphoid nonunion. Hand Clin.

[4] Huene DR, Huene DS (1991). Treatment of nonunions of the scaphoid with the Ender compression blade plate system. J Hand Surg Am Sep.

[5] Leixnering M, Pezzei C, Weninger P (2011). First experiences with a new adjustable plate for osteosynthesis of scaphoid nonunions. J Trauma.

[6] Quadlbauer S, Pezzei C, Jurkowitsch J, Krimmer H, Sauerbier M, Hausner T, Leixnering M (2019). Palmar angular stable plate fixation of nonunions and comminuted fractures of the scaphoid (Article in German). Oper Orthop Traumatol.

[7] Kawamura K, Chung KC (2008). Treatment of scaphoid fractures and nonunions. J Hand Surg Am.

[8] Dodds SD, Williams JB, Seiter M, Chen C (2018). Lessons learned from volar plate fixation of scaphoid fracture nonunions. J Hand Surg Eur Vol.

[9] Jurkowitsch J, Dall'Ara E, Quadlbauer S, Pezzei C, Jung I, Pahr D, Leixnering M (2016). Rotational stability in screw-fixed scaphoid fractures compared to plate-fixed scaphoid fractures. Arch Orthop Trauma Surg.

[10] Mandaleson A, Tham SK, Lewis C, Ackland DC, Ek ET (2018). Scaphoid fracture fixation in a nonunion model: a biomechanical study comparing 3 types of fixation. J Hand Surg Am.

[11] Quadlbauer S, Pezzei C, Beer T (2019). Treatment of scaphoid waist nonunion by one, two headless compression screws or plate with or without additional extracorporeal shockwave therapy. Arch Orthop Trauma Surg.

[12] Goodwin J, Castañeda P, Drace P, Edwards S (2018). A biomechanical comparison of screw and plate fixations for scaphoid fractures. J Wrist Surg.

[13] Goodwin JA, Castañeda P, Shelhamer RP, Bosch LC, Edwards SG (2019). A comparison of plate versus screw fixation for segmental scaphoid fractures: a biomechanical study. Hand (N Y).

[14] Leti Acciaro A, Lana D, Fagetti A, Cherubino M, Adani R (2022). Plate fixation in challenging traumatic carpal scaphoid lesions. Musculoskelet Surg.

[15] Sander AL, Sommer K, Schäf D, Braun C, Marzi I, Pohlemann T, Frank J (2018). Clinical outcome after alternative treatment of scaphoid fractures and nonunions. Eur J Trauma Emerg Surg.

[16] Eng K, Gill S, Hoy S, Shridar V, Van Zyl N, Page R (2020). Volar scaphoid plating for nonunion: a multicenter case series study. J Wrist Surg.

[17] Morgan SD, Sivakumar BS, Graham DJ (2021). Scaphoid plating for recalcitrant scaphoid fractures: a systematic review. J Hand Surg Eur Vol.

[18] Mehling IM, Arsalan-Werner A, Wingenbach V, Seegmüller J, Schlageter M, Sauerbier M (2019). Practicability of a locking plate for difficult pathologies of the scaphoid. Arch Orthop Trauma Surg.

[19] Esteban-Feliu I, Barrera-Ochoa S, Vidal-Tarrason N, Mir-Simon B, Lluch A, Mir-Bullo X (2018). Volar plate fixation to treat scaphoid nonunion: a case series with minimum 3 years of follow-up. J Hand Surg Am.

[20] Putnam JG, DiGiovanni RM, Mitchell SM, Castañeda P, Edwards SG (2019). Plate fixation with cancellous graft for scaphoid nonunion with avascular necrosis. J Hand Surg Am.

[21] Putnam JG, Mitchell SM, DiGiovanni RM, Stockwell EL, Edwards SG (2019). Outcomes of unstable scaphoid nonunion with segmental defect treated with plate fixation and autogenous cancellous graft. J Hand Surg Am.

